# Bleeding hepatocellular adenoma: historical series and outcomes

**DOI:** 10.1590/0100-6991e-20233549-en

**Published:** 2023-06-22

**Authors:** FELIPE ANTÔNIO CACCIATORI, PABLO DUARTE RODRIGUES, PAULO ROBERTO OTT FONTES

**Affiliations:** 1 - Santa Casa de Misericórdia, Serviço de Cirurgia Hepatobiliopancreática e Transplante Hepático - Porto Alegre - RS - Brasil

**Keywords:** Hepatocellular Adenoma, Benign Liver Neoplasm, Abdominal Hemorrhage, Adenoma Hepático, Abdome Agudo Hemorrágico, Neoplasia Benigna do Fígado

## Abstract

**Introduction:**

hepatocellular adenoma - AHC - is a rare benign neoplasm of the liver more prevalent in women at reproductive age and its main complication is hemorrhage. In the literature, case series addressing this complication are limited.

**Methods:**

between 2010 and 2022, 12 cases of bleeding AHC were attended in a high-complexity university hospital in southern Brazil, whose medical records were retrospectively evaluated.

**Results:**

all patients were female, with a mean age of 32 years and a BMI of 33kg/m^2^. The use of oral contraceptives was identified in half of the sample and also half of the patients had a single lesion. The mean diameter of the largest lesion was 9.60cm and the largest lesion was responsible for bleeding in all cases. The presence of hemoperitoneum was documented in 33% of the patients and their age was significantly higher than the patients who did not have hemoperitoneum - 38 vs 30 years, respectively. Surgical resection of the bleeding lesion was performed in 50% of the patients and the median number of days between bleeding and resection was 27 days. In only one case, embolization was used. The relation between ingrowth of the lesions and the time, in months, was not obtained in this study.

**Conclusion:**

it is concluded that the bleeding AHC of the present series shows epidemiological agreement with the literature and may suggest that older patients trend to have hemoperitoneum more frequently, a fact that should be investigated in further studies.

## RESUMO

**Introdução:**

o Adenoma Hepatocelular - AHC - é uma neoplasia benigna rara do fígado associada a mulheres com idade reprodutiva. Sua principal complicação é o sangramento,mas as séries de casos direcionadas a esta intercorrência são limitadas.

**Métodos:**

entre os anos de 2010 e 2022 foram registrados 12 casos de AHC sangrante em um hospital universitário de alta complexidade no sul do Brasil, cujos prontuários foram retrospectivamente avaliados.

**Resultados:**

todos os pacientes eram do sexo feminino, com média de idade de 32 anos e de IMC de 33kg/m^2^. O uso de anticoncepcionais foi identificado em metade da amostra e também metade das pacientes apresentava lesão única. O diâmetro médio da maior lesão foi de 9,60cm e a maior lesão foi responsável pelo sangramento em todos os casos. Hemoperitôneo foi documentado em 33% das pacientes e a idade destas foi significativamente maior do que as pacientes que não apresentaram hemoperitôneo - 38 vs 30 anos, respectivamente. A ressecção cirúrgica da lesão sangrante foi realizada em 50% das pacientes e a mediana de dias entre o sangramento e a ressecção foi de 27 dias. Em apenas um caso foi lançado mão da embolização da lesão. Não se demonstrou correlação entre a taxa de redução das lesões e o passar dos meses, nos casos em que foi adotado tratamento conservador.

**Conclusão:**

conclui-se que aos AHC sangrantes da presente série apresentam concordância epidemiológica com a literatura e pode sugerir que as pacientes com maior idade tendem a apresentar hemoperitôneo mais frequentemente, fato que deve ser investigado em maiores estudos.

## INTRODUCTION

Hepatocellular adenoma (HCA) is a rare benign neoplasm of the liver, with a strong association with the use of oral contraceptives (OC), thus being more prevalent in women of reproductive age. The estimated incidence for these patients is 13 per 100,000/year, as opposed to only 1 per 1,000,000/year for non-users of OCs or women with less than 2 years of exposure to these drugs^1^ ^-^ ^3^.

The diagnosis of HCA is usually based on an incidental finding that triggers the search for a definitive diagnosis, based mainly on imaging studies. Diagnostic certainty can be difficult, requiring the use of specific contrast tests^4^. The importance of differentiating between HCA and other benign liver neoplasms, such as hemangioma and focal nodular hyperplasia, lies in the existence of potential complications exclusively associated with HCA: bleeding and malignant transformation.

Bleeding occurs in up to 27% of cases, being more commonly associated with lesions larger than 5cm^5^. Its occurrence can be intra-lesional, intra-hepatic, or intra-peritoneal, the latter leading to an acute hemorrhagic abdomen, which may result in shock and risk of death^6^.

A recent systematic review found 11 case series published worldwide between 2000 and 2014, totaling 167 cases of bleeding HCA^7^. Another review between 2000 and 2010 considered only series with more than 10 patients and found eight studies, with 143 cases^4^. Thus, the scarcity of large case series is noted, which makes it difficult to extract concise data that support clinical decisions in the face of such a threatening pathology. Therefore, the present study is justified by the need to expand the casuistry of the world literature about bleeding HCAs, to enable treatments guided by solid evidence in the future.

## METHODS

This is a retrospective cohort study, based on secondary data8, developed at a highly complex university hospital located in Southern Brazil, with availability of diagnostic and therapeutic resources necessary for the management of the pathology in question, including interventional radiology methods. The institution’s Ethics Committee was duly consulted and approved the present work, in accordance with the consolidated opinion CAAE 58679922.9.0000.5335, registered in Plataforma Brasil.

We reviewed medical records of patients diagnosed with benign liver neoplasm, ICD D13.4, and who required hospitalization, totaling about 150 records between 2010 and 2022. After individual and sequential analysis of these records, we identified 12 cases of bleeding HCA, which were included in this study.

We collected, tabulated, and analyzed the data using the IBM SPSS software, version 18. We constructed univariate tables to describe the population profile and the frequency of categories in the independent variables. We used bivariate tables to verify the association of outcomes with dependent variables and applied the chi-square test at a 5% significance level^9^ ^-^ ^11^. The nomenclature adopted for referring to the hepatic segments follows the Brisbane terminology^12^.

## RESULTS

All 12 cases of bleeding HCA were female patients. Table 1 describes the sample characteristics, with an average age of 32 years and an average Body Mass Index (BMI) of 33 kg/m^2^. Half of the patients used OC in the 12 months preceding the bleeding. In addition, half of the patients were pregnant at least once and four of the patients were using antihypertensive drugs.

**Table 1:** Sample characteristics.

Variable	n=12	Feature	n=12
Age*	32.95 ± 5.97	Bleeding lesion segment**	
BMI*	33.86 ± 8.04	II	2 (16.70)
OC use in the last 12 months**		III	1 (8.33)
Yes	6 (50.00)	V	4 (33.33)
No	6 (50.00)	VI	3 (25.00)
Previous pregnancies**		VII	2 (16.70)
Yes	6 (50.00)	Subcapsular hematoma**	
No	6 (50.00)	Yes	10 (83.33)
Use of medications**		No	2 (16.67)
Yes	6 (50.00)	Hemoperitoneum**	
No	6 (50.00)	Yes	4 (33.33)
Number of injuries**		No	8 (66.67)
1	6 (50.00)	Hepatic steatosis**	
2	1 (8.30)	Yes	6 (50.00)
3	1 (8.30)	No	6 (50.00)
Variable	n=12	Feature	n=12
5	1 (8.30)	Surgical resection**	
>10	3 (25.00)	Yes	6 (50.00)
Diameter of the largest lesion in cm*	9.60 ± 1.68	No	6 (50.00)
		Residual lesions after 1 year**	
		Yes	6 (50.00)
		No	6 (50.00)

*Values expressed as mean and standard deviation; **Values expressed in absolute numbers and percentiles.

The average diameter of the largest lesion was 9.60 cm and in all cases the lesion responsible for the bleeding was the largest. Half of the patients had only one hepatic adenoma, but a quarter of them had classic adenomatosis, with more than 10 lesions. The most frequently affected liver segment was segment V, in 33% of the patients, and subcapsular hematoma was present in the imaging studies of 83% of the patients. On the other hand, only 33% of the patients had hemoperitoneum.

We verified the presence of hepatic steatosis in half of the patients as per the description of the nuclear magnetic resonance exam and, in cases where there was resection, by the surgical description and/or the anatomopathological study.

Regarding the most prevalent symptoms, six patients complained of pain in the right hypochondrium, five mentioned poorly characterized abdominal pain, three had vomiting, three had fever, one had constipation, and one complained of weight loss. Only two patients knew that they had a hepatic adenoma prior to the bleeding episode. Table 2 presents the laboratory characteristics found upon admission shortly after the bleeding, as well as the intervals in days between the related events.

**Table 2:** Laboratory findings on admission and intervals between events.

Variable	Value
Hemoglobin*	10.44 ± 2.13
Platelets*	374,222 ± 150,541
AST**^10^	35.00 (26.50115.25)
ALT**^7^	71.00 (27.00221.00)
AP**^7^	148.00 (98.00384.00)
GGT**^7^	113.00 (63.00492.00)
LDH*^2^	367.50 ± 16.26
PCR*^6^	215.09 ± 98.44
INR*^9^	1.09 ± 0.09
KTTP*^8^	29.20 ± 28.15
Creatinine*^10^	0.61 ± 0.15
Urea*^9^	21.88 ± 6.90
Days between bleeding and surgery**	27 (9.5180)
Days of hospitalization after surgery**	14 (932)

Statistical analysis showed a significant difference when comparing the mean age in years of patients with hemoperitoneum, 38.75 ± 4.11, with patients without hemoperitoneum, 30.00 ± 4.44 (p=0.013). Other variables were tested and showed no statistical correlation with this outcome. We also found no statistical significance when comparing the occurrence of hemoperitoneum with the segments of the bleeding lesion, with the use of OC, with the occurrence of previous pregnancies, with the previous use of medications, and with the presence of hepatic steatosis. The use of contraceptives did not significantly participate in lesion reduction after bleeding and did not seem to affect the maximum size of the lesions. There was no statistical relationship between BMI and the maximum diameter of the lesions or the rate of reduction, nor with the number of lesions.

Of the 12 patients, four did not undergo any invasive treatment. Among the seven patients who were operated, one underwent only lavage of the peritoneal cavity due to hemoperitoneum, without approaching the hepatic bleeding and the lesion itself. Of the six patients who submitted to adenoma resection, there were: one enucleation of a single lesion in segment VI, one hepatectomy in segments V and VI, one resection in segment VIII, one right anterior sectorectomy, one left lateral sectorectomy, and one resection of segments V, VI, and VII.

Only one patient was treated by gelfoam embolization. This woman presented a single lesion measuring 8.7cm in segment VIII, with hemoperitoneum and admission hemoglobin of 9.4g/dL. Embolization was performed six days after the bleeding and in the control Computed Tomography (CT) after three months, the lesion had regressed to 4.2cm, not being submitted to surgical resection in the follow-up.

Regarding complications after treatment, we identified no deaths in the series and one patient required prolonged hospitalization: a 36-year-old patient, BMI 27kg/m^2^, who was clinically stable and had five lesions, the largest measuring 13cm in segment V, with subcapsular hematoma but without hemoperitoneum, hemoglobin at admission of 14.6g/dL and other normal tests, was released after initial hospital observation of seven days for outpatient follow-up. A CT was repeated after two months, displaying regression of the largest lesion, then measuring 3.9cm in the largest axis. Hepatectomy was performed six months after the bleeding episode, resulting in the resection of segments V, VI, and VII, where most of the lesions were concentrated. This patient evolved well in the first postoperative days, but was readmitted after discharge due to subphrenic collection, requiring percutaneous drainage and prolonged hospitalization, for about 60 days.

All operated patients had an anatomopathological study confirming the diagnosis of HCA, but none had an immunohistochemical study. At follow-up, one patient was diagnosed with high-grade serous ovarian carcinoma six years after the adenoma bleeding episode. Another became pregnant years after the bleeding, without complications.

Of the six patients without lesions after one year of bleeding, five also belong to the group that underwent surgical resection and one showed spontaneous, complete regression, without surgery or embolization: she had a 10cm lesion in segment II, which was no longer identified after the bleeding episode in follow-up exams.

**Figure 1:**
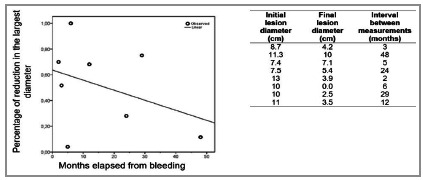
Percentage and absolute numbers of lesion reduction over the months.

When analyzing the rate of reduction in the lesions’ size from the date of the first image, we found no statistical correlation between the size of the lesions and the rate of reduction, nor between the rate of reduction and the time interval in months. Figure 1 presents these data, demonstrating that while some lesions reduced more than 60% in their largest diameter within 15 months, others showed minimal reduction after 48 months. The average initial diameter was 9.86 ± 1.93cm, the average final diameter was 4.57 ± 3.01cm, the average decrease in the largest diameter was 5.28 ± 3.74cm, and the average interval between the exams was 16.12 ± 16.29 months.

## DISCUSSION

The clinical presentation of ruptured hepatic adenoma is variable. Symptoms of sudden and progressive abdominal pain, with frank peritonitis when there is free blood in the peritoneal cavity, is the classic picture, although its occurrence is not universal. Cases of intra-tumoral or intra-hepatic rupture generate more restricted symptoms, such as continuous and progressive pain in the right hypochondrium, without relieving factors or relation with food or position^13^ ^-^ ^15^. There is even a description of bleeding from a hepatic adenoma that occurred during the early postoperative period of a patient with rupture of another adenoma^16^. In the present series, in addition to the symptoms mentioned in the literature, we identified cases of feverish symptoms and even weight loss.

It is a consensus that in cases of hepatic tumor bleeding, the first management concerns hemodynamic resuscitation and treatment of coagulation disorders^7^. The patient who is stable and without extrahepatic bleeding should be submitted to a better investigation of the lesion, preferably with Magnetic Resonance Imaging (MRI), to elucidate eventual diagnostic doubt in relation to adenoma versus hepatocellular carcinoma^17^. The presence of liver cirrhosis should make the surgeon even more aware of the possibility of a malignancy.

Interventional radiology treatment by selective hepatic artery embolization is the treatment of choice for patients with bleeding HCA and hemodynamic stability^7^ ^,^ ^13^, which may or may not be followed by lesion resection in a more favorable surgical scenario. In cases of hemodynamic instability, the surgical approach is an emergency, usually with hepatic packing and gross hemostasis for damage control, followed by resuscitation in an intensive care environment for subsequent surgical reapproach^7^. The use of hemostatic maneuvers such as Pringle’s greatly helps to contain the hemorrhage, and digital dissection for rapid removal of the lesion, as proposed by Lin^18^ ^,^ ^19^, may be necessary in these cases. In the 12 cases reported herein, no patient had hemodynamic instability, so all were investigated with MRI, and in no case was there any doubt as to the possibility of malignancy. The median of days between bleeding and surgery, when performed, was 27 days, demonstrating the non-urgent character of the resections.

A series of 167 patients from a North American series, comprising individuals with a mean age of 39.8 ± 12.9 years and a mean BMI of 28.6 ± 6.5kg/m^2^, reported that 27 patients had bleeding, of which all women. The BMI of the group that bled did not differ significantly from those that did not. Among the patients who bled, 48.2% had a single lesion, and 37% had 10 or more. Management of patients who bled included resection in 63% of patients, while 22.2% were treated with a combination of surgical resection and embolization or radioablation, and 7.4% were treated conservatively. It is interesting to mention that two patients in this sample underwent liver transplantation and 1.2% had malignant lesions^15^.

A French case series found 56 patients with bleeding HCA between 1990 and 2013. The median age of the sample was 39 years, median BMI 24kg/m^2^, and median hemoglobin at admission, 10g/dL^20^. In the present series, patients were also all females, but with a younger age, 32 years on average, and a higher BMI, 33kg/m^2^ on average.

The literature suggests that lesions located in segments II and III have a greater tendency to bleed and that exophytic lesions bleed more than subcapsular ones, which in turn bleed more than intrahepatic ones. Another characteristic that increases the risk of bleeding is the visualization of the supplying artery, whether peripheral or central^21^. The cases reported here were mostly located in the right hepatic lobe, especially in segment V, and the detailed evaluation of the lesions’ imaging characteristics was beyond the proposed scope.

The evolution of the study of molecular subtypes allowed the identification of factors associated with a higher risk of complications, specifically hemorrhage and malignant transformation. In some series, the bleeding rate in women at risk, considering those with mutation in exons 7 or 8 of beta-catenin, reaches 26-27%^5^ ^,^ ^6^, but classically the literature considers the rate to be around 14-16% of bleeding, especially in tumors larger than 5cm^5^ ^,^ ^15^ ^,^ ^22^. In lesions smaller than 5cm and with beta-catenin mutation, however, the risk of bleeding would be increased even with the reduced lesion size^5^.

A historical series of 83 cases of bleeding HCA treated between 1984 and 2020 in a French hospital, in which 78 patients were operated on, found no mortality associated with the procedure, but serious complications occurred in 11.5%. In the same series, review and immunohistochemistry of the lesions showed an association between bleeding and b-catenin mutation subtypes in exons 7 and 8, in addition to sonic hedgehog mutations, lesion size, and chronic alcohol consumption^23^. The absence of genetic tests and immunohistochemistry in the series presented here did not allow the evaluation of the molecular factors seen in these other articles.

A series of 23 cases of patients with massive intrahepatic hemorrhage or hemoperitoneum showed 100% female patients, with a median age of 34 years and a median lesion size of 7.6cm. OC use was identified in 82.6% of them. The median hemoglobin value at admission was 12.24g/dL and the median length of stay was 13 days. None of them had a previous diagnosis of HCA and 65.2% had hemodynamic stability. Conservative treatment was indicated for 56.5% of the patients and 39.1% of them were initially submitted to selective angioembolization in the acute phase, the others being operated upon admission. As for complications, two patients who underwent embolization or resection had intrahepatic abscess, requiring drainage and prolonged hospitalization^17^.

The rebleeding rate in that series was 4.3% and the bleeding occurred three months after the initial hemorrhage. After 36 months of follow-up, all tumors regressed, with a reduction in the median size from 7.6cm to 2.5cm in the series, elective resection being performed only in patients who subsequently had lesions larger than 5cm or who expressed a desire to become pregnant^17^. These findings corroborate ours, especially regarding the similarity of the ages, the dimensions of the bleeding lesions, and the hemoglobin values at admission.

We found no work in the literature that reported the rate of reduction of lesions over the months in cases in which surgical resection was postponed. This study demonstrated no correlation between the rate of lesion reduction after bleeding and initial diameter or time. This represents the unpredictability of the evolution of these lesions after the hemorrhagic episode, so that further studies could verify these findings.

## CONCLUSION

The scarcity of large case series on hepatocellular adenoma with acute bleeding and its management and outcomes highlights the need for further studies in this area. The relationship between hormonal factors, such as the use of oral hormonal contraceptives, obesity, female sex, and the use of OCs seems to be evident, but their influence on the occurrence of bleeding still needs to be better characterized. In the present series, the occurrence of hemoperitoneum was seen more in patients with higher age, but the clinical implications of this data are not unequivocal.

Late management in cases of patients who presented bleeding and were treated without surgical interventions certainly requires further studies. There is no consensus on the indication of resection for lesions that regress to dimensions smaller than 5cm after a bleeding episode, and there are authors who only observe this type of lesions^20^. One could infer that the mutations associated with the bleeding are also associated with malignant transformation, which would justify liberal resection in such cases. However, no clear evidence was found for this recommendation.

The findings of this study regarding the non-predictable behavior of lesions after bleeding, in terms of their diameter reduction, should serve as a basis for future studies with larger samples and statistical power to verify such occurrences.
